# Expression of claudin‐18.2 in cholangiocarcinoma: a comprehensive immunohistochemical analysis from a German tertiary centre

**DOI:** 10.1111/his.15407

**Published:** 2024-12-27

**Authors:** Maximilian N Kinzler, Steffen Gretser, Falko Schulze, Katrin Bankov, Nada Abedin, Wolf O Bechstein, Fabian Finkelmeier, Stefan Zeuzem, Henning Reis, Peter J. Wild, Dirk Walter

**Affiliations:** ^1^ Goethe University Frankfurt Medical Clinic 1, University Hospital Frankfurt am Main Germany; ^2^ Goethe University Frankfurt Dr. Senckenberg Institute of Pathology, University Hospital Frankfurt am Main Germany; ^3^ Department of Pediatric Oncology and Hematology Charité–Universitätsmedizin Berlin, Corporate Member of Freie Universität Berlin and Humboldt‐Universität zu Berlin Berlin Germany; ^4^ Department of General, Visceral, Transplant and Thoracic Surgery University Hospital, Goethe University Frankfurt Frankfurt am Main Germany; ^5^ Frankfurt Cancer Institute (FCI), Goethe University Frankfurt Frankfurt am Main Germany; ^6^ Frankfurt Institute for Advanced Studies (FIAS) Frankfurt am Main Germany

**Keywords:** biomarker, cholangiocarcinoma, CLDN18, surgical oncology, tight junctions

## Abstract

**Aims:**

Anti‐claudin‐18.2 (CLDN18.2) therapy was recently approved for the treatment of gastric or gastro‐oesophageal junction adenocarcinoma. The aim of the present study was to investigate the expression of CLDN18.2 in cholangiocarcinoma (CCA) to determine whether there is a subgroup of patients who might also benefit from anti‐CLDN18.2 therapy.

**Methods and results:**

A tissue microarray (TMA) cohort of all CCA patients who underwent surgical resection with curative intent between August 2005 and December 2021 at University Hospital Frankfurt were immunohistochemically evaluated using the VENTANA^®^ CLDN18 (43‐14A) antibody. Tumour positivity for CLDN18.2 was determined as follows: ≥ 75% of tumour cells with moderate‐to‐strong CLDN18 membranous staining. In total, 160 patients with surgically resected CCA were suitable for immunohistochemistry (IHC) analysis. Of the patients, 13.1% (*n* = 21) showed moderate to strong membranous staining of VENTANA^®^ CLDN18 antibody, while 86.9% (*n* = 139) were negative. Subtype analysis revealed strong differences in CLDN18 expression. Positive staining of CLDN18 could be observed in 26.5% (*n* = nine of 34) and 7.4% (*n* = seven of 95) of the perihilar (pCCA) and intrahepatic (iCCA) subgroup, respectively. CCA patients with CLDN18 expression had a more frequently intraoperative finding of distant metastasis (*P* = 0.002), lymph node metastasis (*P* = 0.008) and positive perineural invasion (Pn1) status (*P* = 0.022).

**Conclusions:**

The present study suggests that a subset of patients with CCA exhibited a marked expression of CLDN18.2. These findings underline the need to perform a clinical study evaluating the efficacy of anti‐CLDN18.2 therapy in patients suffering from CCA.

**Abbreviations**
CCA: Cholangiocarcinoma
CLDN18.2: Claudin‐18.2
CI: Confidence Interval
dCCA: Distal Cholangiocarcinoma
EMA: European Medicines Agency
FDA: Food and Drug Administration
HR: Hazard Ratio
IHC: Immunohistochemistry
IQR: Interquartile Range
iCCA: Intrahepatic cholangiocarcinoma
LD‐iCCA: Large duct‐type intrahepatic Cholangiocarcinoma
OS: Overall Survival
PDAC: Pancreatic Ductal Adenocarcinoma
pCCA: Perihilar Cholangiocarcinoma
ROI: Regions of Interest
SD‐iCCA: Small duct‐type intrahepatic Cholangiocarcinoma
TMA: Tissue Microarray
UCT: University Cancer Center Frankfurt

## Introduction

Cholangiocarcinoma (CCA) is an epithelial neoplasm arising from intrahepatic as well as extrahepatic bile ducts. Despite the rare occurrence, the incidence is increasing.^1^ Standard of care in the palliative setting is a combination of chemotherapy and immune check‐point inhibition with a median overall survival of less than 13 months.^2^, ^3^ Due to this devastating outcome, identification of new potential therapeutic options is of utmost importance to improve prognosis of these patients.

Tight junctions molecules have multiple functions in maintaining intercellular adhesion. These proteins have been observed to be altered or dysfunctional in many types of cancer.^4^ This alteration can lead to modulation of cytoskeletal elements and signalling molecules, resulting in a loss of regulated cell migration and proliferation.^5^ Claudins belong to the major components of tight junctions forming associations with their counterparts on adjacent cells.^6^ These molecules have attracted attention in carcinogenesis, as up‐regulation and mislocalisation of the splice variant claudin‐18.2 (CLDN18.2) was observed in various neoplasms. Its expression in normal tissue is strictly restricted to gastric mucosa and is retained during malignant transformation in the case of gastric cancer. CLDN18.2 was also found later to be ectopically activated in other malignancies.^7^

Recently, the monoclonal antibody Zolbetuximab, which targets CLDN18.2, was approved by the Food and Drug Administration (FDA) and European Medicines Agency (EMA) in combination with chemotherapy for the treatment of locally advanced, unresectable or metastatic gastric or gastro‐oesophageal junction adenocarcinoma following the results of the SPOTLIGHT study.^8^ This study raised the question of whether this therapeutic approach might also be applicable to other tumour entities. To our knowledge, data on the expression of CLDN18.2 in CCA using the same antibody as in the SPOTLIGHT trial is missing to date.

Therefore, the aim of the present study was to investigate the expression of CLDN18.2 in CCA with the VENTANA^®^ CLDN18 (43‐14A) antibody, which was used in the SPOTLIGHT study, to determine whether a subset of CCA patients could potentially benefit from anti‐CLDN18.2 antibody therapy.

## Materials and methods

### Study population

The study builds upon the patient population and data base of a previously reported tissue microarray (TMA) cohort of CCA patients who were surgically resected at Frankfurt University Hospital between August 2005 and December 2021.^9^, ^10^ Histopathological confirmation was assessed by expert pathologists of the Dr Senckenberg Institute of Pathology, University Hospital Frankfurt. Small duct (SD‐iCCA)‐ and large duct (LD‐iCCA)‐ type intrahepatic cholangiocarcinoma (iCCA) examined in the present study were analysed histomorphologically and immunohistochemically and assigned to the respective subtype in our previously published work.^11^ Tissue samples used in this study were provided by the University Cancer Center Frankfurt (UCT). Written informed consent was obtained from all patients and the study was approved by the Institutional Review Boards of the UCT and the Ethical Committee at the University Hospital Frankfurt (project number: SGI‐13‐2018).

### Immunohistochemistry (IHC) analysis

**Figure 1:**
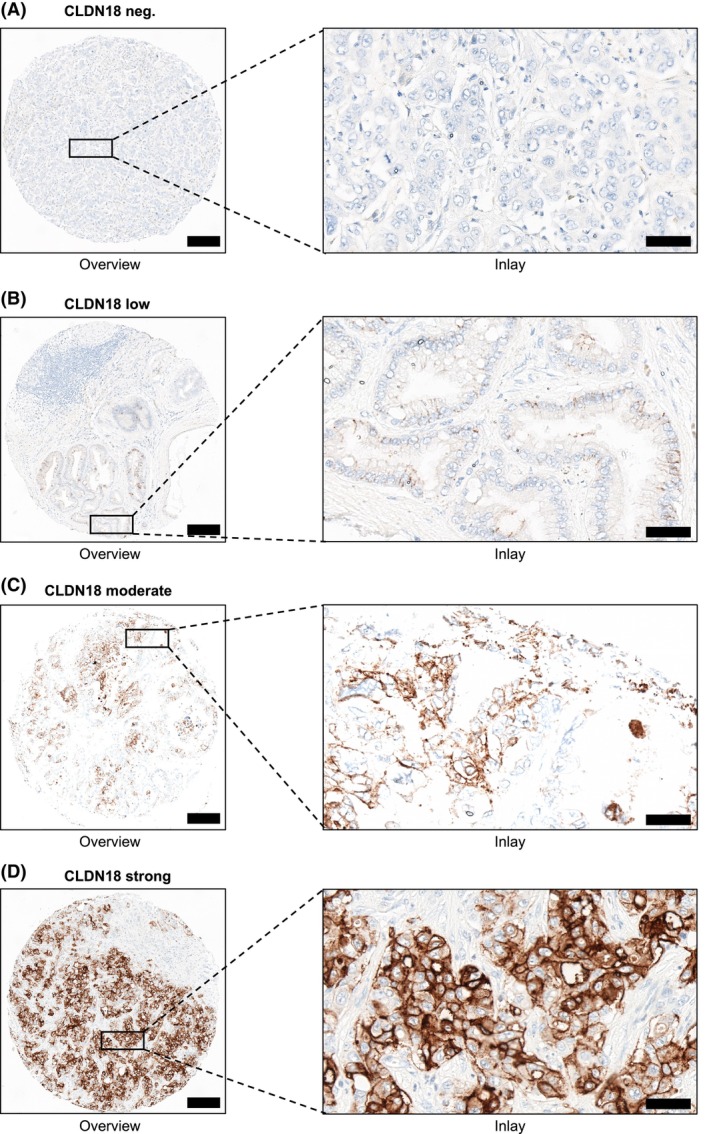
Representative images of claudin‐18.2 (CLDN18) expression in cholangiocarcinoma (CCA). Representative immunohistochemistry of negative (**A**), low (**B**), moderate (**C**) and strong (**D**) expression of VENTANA^®^ CLDN18 staining in tissue microarray (TMA) cores of CCA patients. Scale bars: 200 μm for overview and 50 μm for inlay. [Color figure can be viewed at wileyonlinelibrary.com]

TMA construction was performed as previously reported.^9^ In short, annotations of defined regions of interest (ROI) containing representative tumour area were set to a core diameter of 1.0 mm. In our study, one TMA core per case was investigated immunohistochemically. Staining of VENTANA^®^ CLDN18 (clone: 43‐14A ready‐to‐use, incubation time: 16 min; Roche, Basel, Switzerland) and HER2/*neu* (polyclonal antibody concentrate, dilution 1:400, incubation time 30 min; Agilent, Santa Clara, CA, USA) was conducted. Immunohistochemical evaluation of CLDN18.2 was performed manually, and cellular positivity was defined as previously published: either strong or intermediate circumferential staining, or only strong partial staining.^12^ Tumour positivity for CLDN18.2 (≥ 75% of tumour cells with moderate‐to‐strong CLDN18 membranous staining) was determined analogously to the SPOTLIGHT and GLOW trials.^8^, ^13^ Figure 1 displays representative images showing negative, low, moderate and strong membranous staining of VENTANA^®^ CLDN18 in CCA tissue, respectively.

### Statistical analysis

We compared baseline clinicopathological characteristics between patients with absence and presence of VENTANA^®^ CLDN18 expression. Categorial variables are presented as frequencies and percentages and continuous variables are shown as means with standard deviations. Categorial and continuous variables were compared using Student's *t*‐test and χ^2^ test, respectively. Overall survival (OS) was defined as the time of onset of disease until death. Patients alive or lost to follow‐up were treated as censored observation. Survival was compared using the log‐rank test. The Kaplan–Meier curve for survival was derived to visualise the comparison between CCA patients with positive and negative CLDN18 expression. Cox regression analysis for CLDN18 was performed to assess risk factors impacting patient survival. The significance level was set to *P* < 0.05. All data were analysed with SPSS version 27 (IBM, Armonk, NY, USA) statistical software and GraphPad Prism version 10.2.3.

## Results

### Clinical characteristics

In total, 160 patients with surgically resected CCA in our tertiary hospital were suitable for IHC analysis after TMA construction. HER2‐status was assessed by IHC, and all patients were HER2‐negative (score 0). The median age was 66 years [interquartile range (IQR) = 60–73]. In total, 65% were male (*n* = 105) and 35% were female (*n* = 56). Among the patients, 59.3% were diagnosed with iCCA (*n* = 95; SD‐iCCA: *n* = 68/LD‐iCCA: *n* = 27), 21.3% with perihilar (pCCA; *n* = 34) and 19.4% with distal (dCCA; *n* = 31) cholangiocarcinoma. CCA patients with CLDN18 expression more frequently had intraoperative findings of distant metastasis (*P* = 0.002), lymph node metastasis (*P* = 0.008) and positive perineural invasion (Pn1) status (*P* = 0.022). In contrast, pathological findings of lactate dehydrogenase in serum were detected more frequently in patients without CLDN18 expression (*P* = 0.036). CCA patients with positive or negative staining of CLDN18 did not differ in any other clinicopathological findings (Supporting information, Table S1).

### Immunohistochemical assessment of VENTANA ^®^ CLDN18 in CCA

In total, 13.1% (*n* = 21) of the enrolled patients showed moderate‐to‐strong membranous staining of VENTANA^®^ CLDN18 antibody in more than 75% (75–100%, median = 90%) of the tumour cells while 86.9% (*n* = 139) were negative (Supporting information, Figure S1). Of the 139 negative cases, 20.1% (*n* = 28) exhibited a partial positivity (5–60%, median 10%), predominantly showing complete or partial moderate‐to‐strong membranous staining, while three cases displayed granular staining. To evaluate potential heterogeneity in CLDN18 expression, we performed staining on the corresponding whole‐slide images of representative TMA cores. However, it is important to note that no cases have been reclassified, but that the percentage of moderate or strong ratings may vary (Supporting information, Figure S2). In five of nine cases, discrepancies were observed between the scoring of the TMA cores and the whole‐slide counterparts. Within the group displaying ≥ 75% CLDN18 expression, discrepancies were noted in two of three cases, with differences ranging from 15 to 20% (average 13%). In contrast, all three cases in the < 75% CLDN18 expression group showed variability, with differences spanning from 10 to 50% (average 27%).

### Expression of VENTANA ^®^ CLDN18 among CCA subtypes

**Figure 2:**
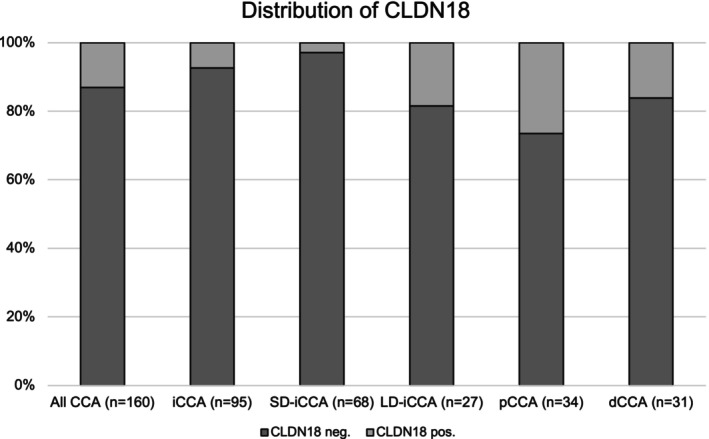
Distribution of claudin‐18.2 (CLDN18) expression among cholangiocarcinoma (CCA) subtypes. dCCA, distal cholangiocarcinoma; iCCA, intrahepatic cholangiocarcinoma; LD‐iCCA, large duct‐type intrahepatic cholangiocarcinoma; pCCA perihilar cholangiocarcinoma; SD‐iCCA, small duct‐type intrahepatic cholangiocarcinoma.

As we detected a marked proportion of CCA patients with CLDN18 expression, we further analysed potential differences between the CCA subtypes. In total, 7.4% (*n* = seven) of iCCA patients showed CLDN18 expression while 92.6% (*n* = 88) were negative. Of note, only a minority of 2.9% (*n* = two) in the SD‐iCCA subcohort showed positive staining of CLDN18, while 18.5% (*n* = five) of LD‐iCCA expressed membranous CLDN18. Next, the presence of CLDN18 expression could be observed in 26.5% (*n* = nine of 34) of the perihilar cholangiocarcinoma (pCCA) subgroup, while 16.1% (*n* = five of 31) were positive in distal cholangiocarcinoma (dCCA). Distribution of membranous expression of VENTANA^®^ CLDN18 among the different CCA subtypes is shown in Figure 2.

### Impact of CLDN18 expression on overall survival

Because membranous CLDN18 staining has been associated in our cohort with more aggressive tumour biology, we investigated the impact of CLDN18 expression on survival. However, the Kaplan–Meier curve revealed no difference in OS between CCA patients with presence [47.74 months, 95% confidence interval (CI) = 24.46–71.03 months] or absence (43.85 months, 95% CI = 33.24–54.46 months) of moderate‐to‐strong membranous CLDN18 expression (*P* = 0.713; Supporting information, Figure S3). In line with this, univariate Cox regression revealed that CLDN18 did not serve as a significant risk factor for OS in our study [hazard ratio (HR) = 0.907, 95% CI = 0.534–1.54, *P* = 0.717].

## Discussion

Systemic treatment of advanced gastric cancer was recently extended with the approval of Zolbetuximab, which could demonstrate relevant efficacy in the SPOTLIGHT study.^8^ The aim of this study was to determine the presence and degree of CLDN18.2 expression in CCA using the same antibody as in the pivotal study. To our knowledge, this study is the first to investigate this clinically relevant topic.

In the SPOTLIGHT study, patients were defined as CLDN18.2‐positive if ≥ 75% of tumour cells showed moderate‐to‐strong membranous CLDN18 staining using the VENTANA^®^ CLDN18 (43‐14A) RxDx assay. Thereby, 38% of the patients with gastric cancer or cancer of the gastro‐oesophageal junction fulfilled the criteria. In the present study, we could show that 13.1% of our patient cohort were positive for CLDN18.2. This indicates that a considerable proportion of patients with CCA might qualify for potential treatment with CLDN18.2‐directed antibodies. Notably, this portion is lower (7.4%) than the already approved options of targeted therapy against *IDH1* and *FGFR2*, which occur in 14–20% of patients with iCCA.^14^, ^15^ However, subgroup analysis of the current study revealed that CLDN18.2 expression predominantly occurred in pCCA, which was shown to have the highest proportion of positivity for CLDN18.2. This is of high clinical interest, as targeted options are most frequently lacking for this subgroup. Moreover, within the iCCA group, the large duct‐type displayed CLDN18.2 positivity more frequently than the small duct‐type. It is known that these subtypes differ in location within the liver as well as aetiology and even prognosis.^11^, ^16^, ^17^ While *IDH1* mutations and *FGFR2* fusions occur mainly in the small duct‐type, this study is the first, to our knowledge, to detect a potential therapeutically relevant biomarker occurring more frequently in the large duct‐type.^18^, ^19^ Of note, CLDN18 expression did not affect patient survival in our study. Interestingly, one study demonstrated increased OS rates in surgically resected pancreatic ductal adenocarcinomas (PDAC) with high CLDN18 expression.^20^ However, moderate‐to‐strong membranous expression of CLDN18 was observed in 23.5–54.6% of PDAC cases,^20^, ^21^, ^22^ and high CLDN18 expression was not associated with prognosis in the study by Park *et al*.^22^ Notably, the three studies did not use the VENTANA^®^ CLDN18 (43‐14A) assay, limiting comparability to our data.

Data on CLDN expression in CCA using antibodies different from those in the current study are very limited. In 2020 Li *et al*. observed a low to moderate expression in eight of 12 patients, but no sample with high expression.^23^ Shinozaki *et al*. came to different results, as they observed an expression of CLDN in 43% of iCCA (*n* = 83) and even 90% of extrahepatic CCA (*n* = 99).^24^ Notably, the antibodies used in these studies were directed against CLDN18, but not CLDN18.2‐specific. In contrast, Hong *et al*. observed one of 16 samples of CCA to be positive using an antibody directed against CLDN18.2.^25^ However, this antibody is not the one used in the SPOTLIGHT study and the number of cases was limited, making it difficult to draw conclusions. Importantly, the VENTANA^®^ CLDN18 (43‐14A) assay detects both CLDN18.1 and CLDN18.2 isoforms, which is also a main limitation of the present study. In accordance with recently published studies,^8^, ^13^, ^26^ positive staining of CLDN18 was determined as CLDN18.2 positivity as CLDN18.2 is the dominant isoform observed in gastric and gastro‐oesophageal junction adenocarcinoma, whereas the CLDN18.1 isoform was shown to be insignificant.^27^ However, future studies investigating the expression of CLDN18.2‐specific antibodies in CCA patients are warranted. In line with Hong and colleagues, we recommend the implementation of a standardised antibody and well‐defined pathological criteria for detecting CLDN18 expression to support its use as a novel clinical biomarker.

To interpret the results of this study correctly, it needs to be considered that these are data from a single centre with a mainly caucasian cohort. Thus, data from Asian cohorts are still awaited. Importantly, studies evaluating CLDN18 expression in biopsies of palliative CCA patients are still warranted as we analysed surgically resected specimens, representing a major limitation of our study. Next, the sample size for p/dCCA was limited and generalisability may not be presumed for this subcohort. As we analysed data from a large TMA cohort, it should be considered that TMA cores only represent limited areas of tumour tissue.

## Conclusion

In conclusion, the present study is the first to provide a comprehensive overview of expression of CLDN18.2 in CCA using the VENTANA^®^ CLDN18 (43‐14A) assay. It shows that a subset of patients with CCA exhibit a marked expression of CLDN18.2. These findings underline the need to perform a clinical study evaluating the efficacy of anti‐CLDN18.2 therapy in patients suffering from CCA.

## Conflicts of interest

F.F. received travel support from Ipsen, Abbvie, Astrazeneca and speaker/advisory fees from AbbVie, MSD, Ipsen, Astrazeneca, Roche, BMS; S.Z. received consultancy and/or speaker's bureau, Abbvie, BioMarin, Boehringer Ingelheim, Gilead, GSK, Ipsen, Madrigal, MSD/Merck, NovoNordisk, SoBi and Theratechnologies; H.R., advisory board of Bristol‐Myers Squibb and Roche, received honoraria from Roche, Bristol‐Myers Squibb, Janssen‐Cilag, Novartis, Astra Zeneca, MCI, CHOP GmbH, Sanofi, Boehringer‐Ingelheim, GlaxoSmithKline, Merck and Diaceutics, received travel support from Philips, Roche and Bristol‐Myers Squibb and received grants from Bristol‐Myers Squibb; P.J.W. received consulting fees and honoraria for lectures by Bayer, Janssen‐Cilag, Novartis, Roche, MSD, Astellas Pharma, Bristol‐Myers Squibb, Thermo Fisher Scientific, Molecular Health, Guardant Health, Sophia Genetics, Qiagen, Eli Lilly, Myriad, Hedera Dx and Astra Zeneca; research support was provided by Astra Zeneca. The authors declare that there is no relationship relevant to the manuscripts’ subject. All other authors declare no conflicts of interest.

## Supporting information


**Figure S1.** Flowchart of screening, enrolment, and allocation.

**Figure S2.** Representative images of CLDN18 expression in TMA cores and corresponding whole slides.

**Figure S3.** Kaplan–Meier curve for overall survival in CCA patients with presence and absence of membranous CLDN18 expression.

**Table S1.** Baseline characteristics. Positive M1 status reflects an intraoperative finding of M1 situation (e.g., distant lymph node metastasis) that was not known before surgery.

## Data Availability

The data sets used and/or analysed during the current study are available from the corresponding author upon reasonable request.
