# Supplementary figures and images for: Expression of claudin‐18.2 in cholangiocarcinoma: a comprehensive immunohistochemical analysis from a German tertiary centre
Source: Histopathology. 2024 Dec 27;86(4):640–6. doi: 10.1111/his.15407 (PMC11791722; doi:10.1111/his.15407)

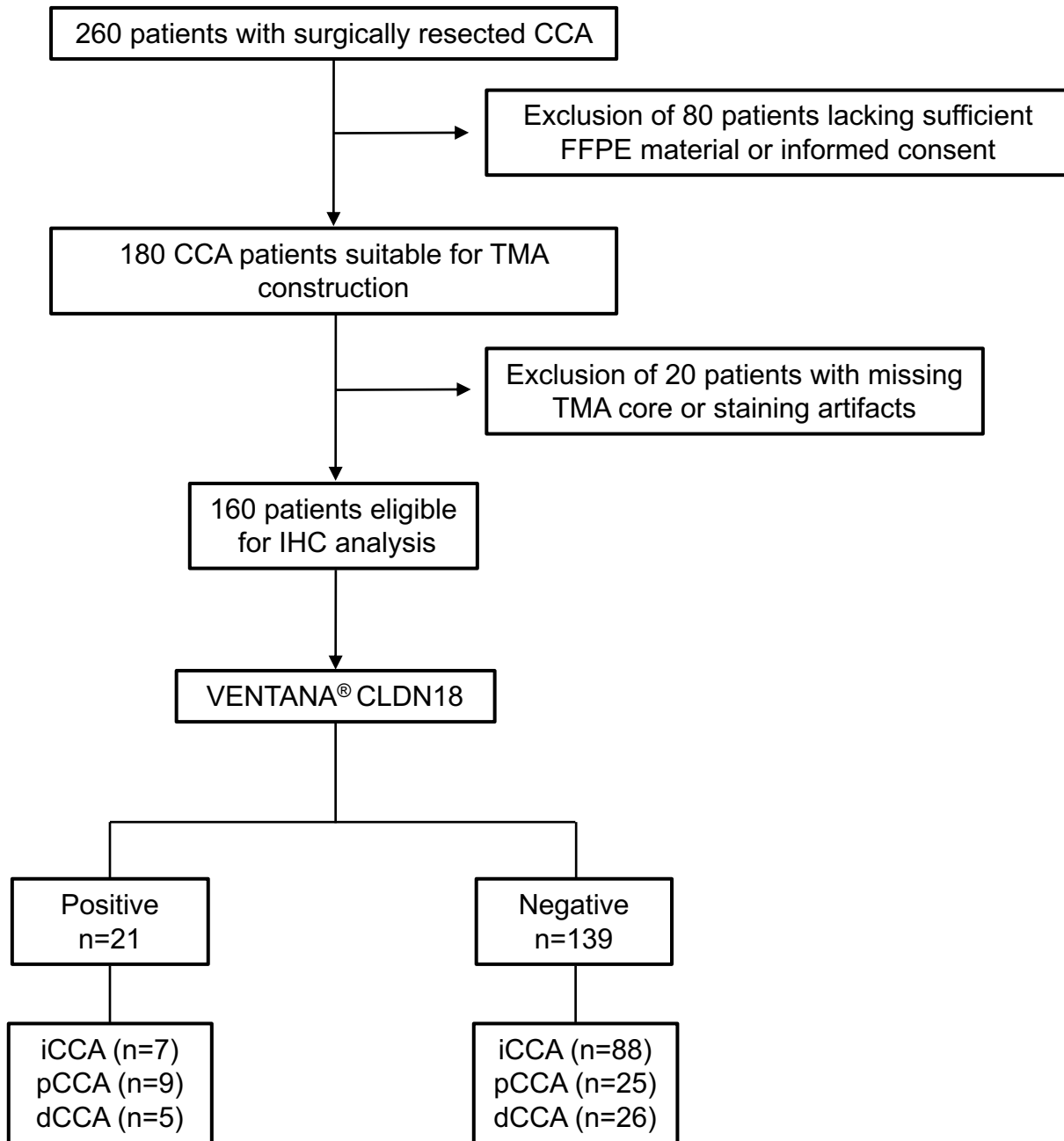

Supplement: Supplementary file 1 — Figure S1. Flowchart of screening, enrolment, and allocation. [file HIS-86-640-s003.pdf]

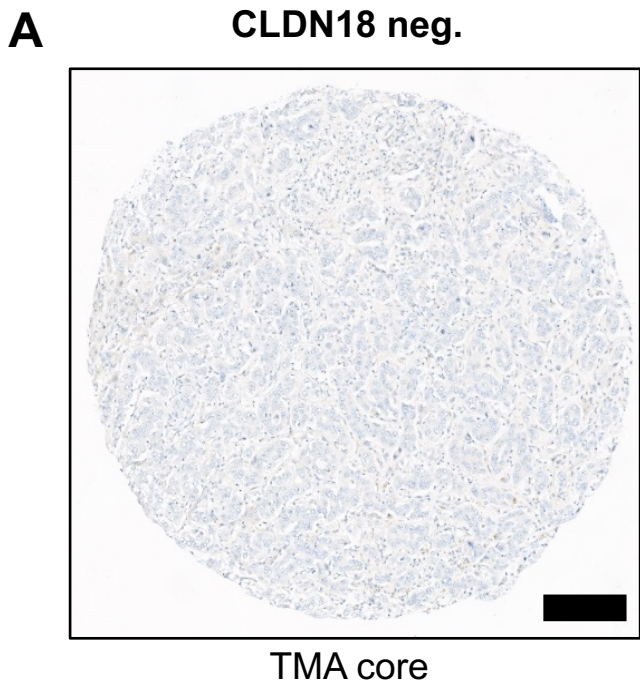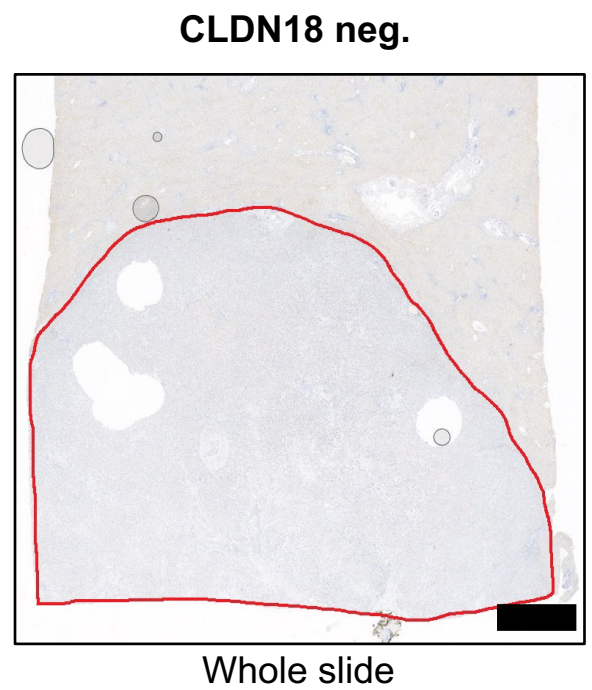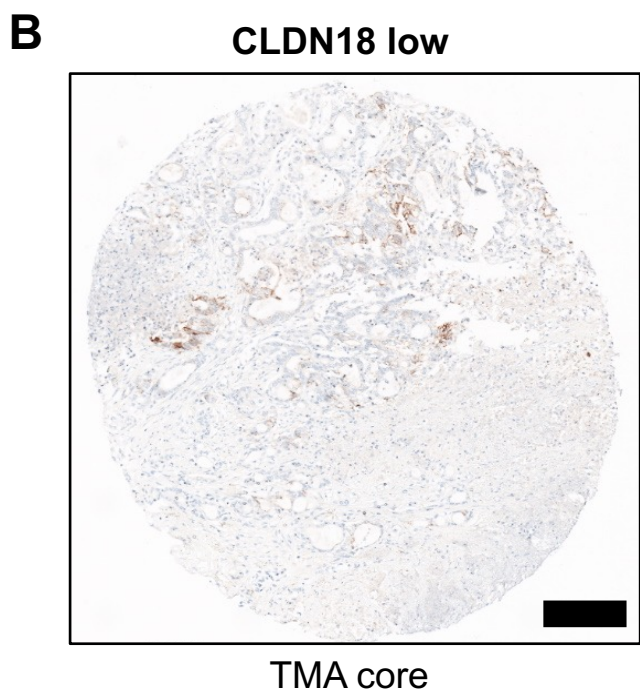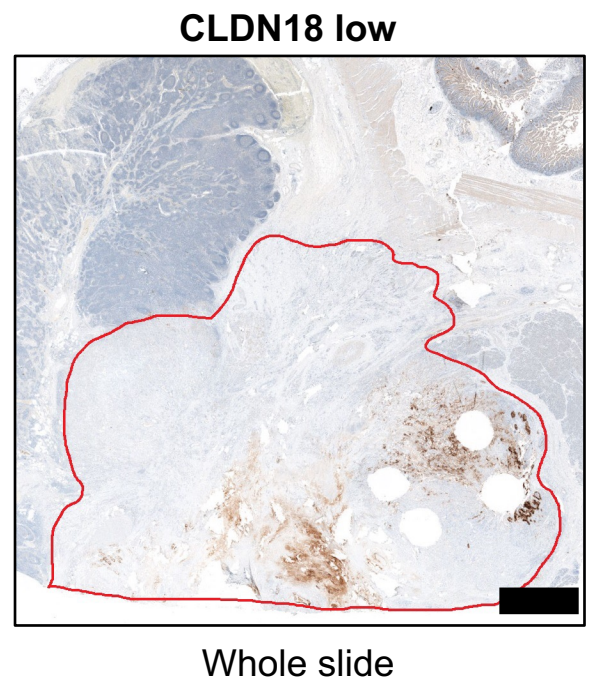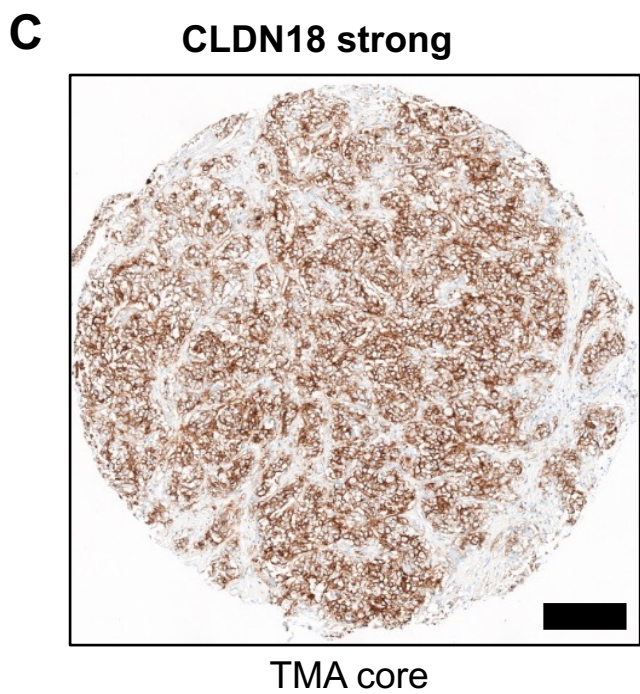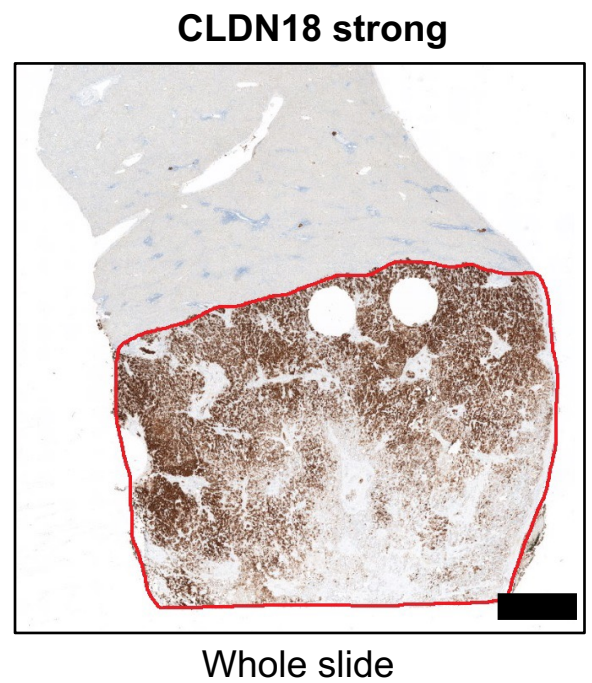

Supplement: Supplementary file 2 — Figure S2. Representative images of CLDN18 expression in TMA cores and corresponding whole slides. [file HIS-86-640-s002.pdf]

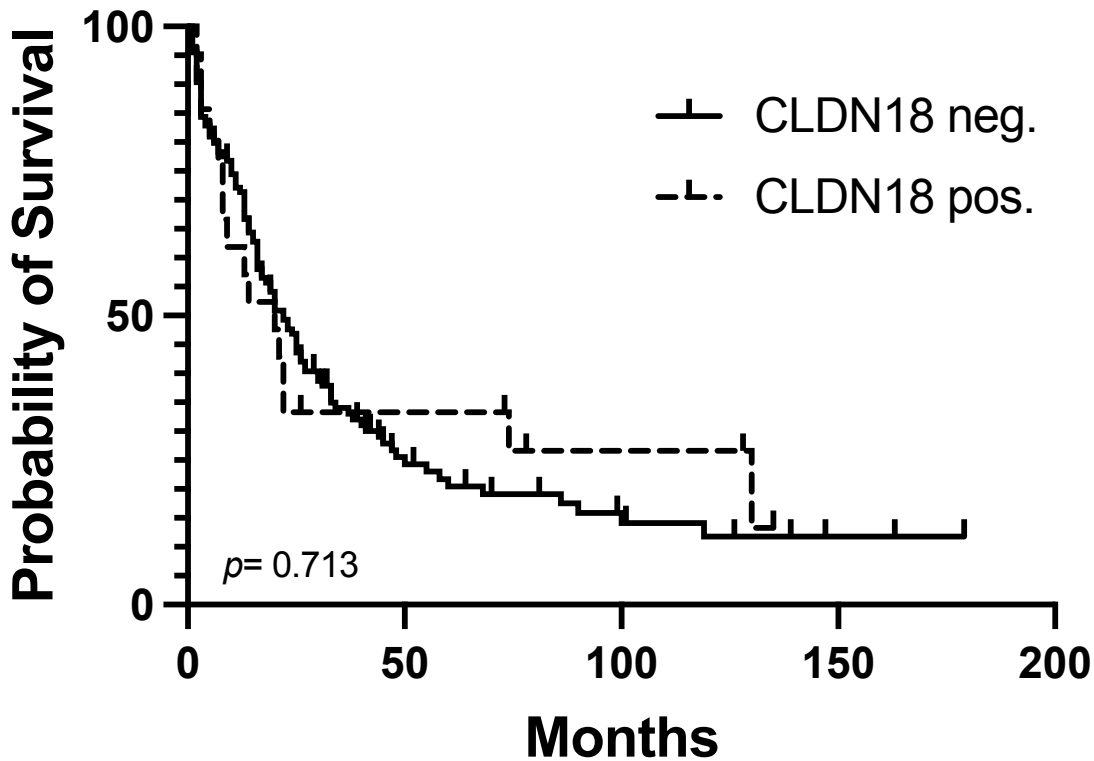

Number at risk

CLDN18 neg. 139

20

8

2

0

CLDN18 pos. 21

6

3

0

0

Supplement: Supplementary file 3 — Figure S3. Kaplan–Meier curve for overall survival in CCA patients with presence and absence of membranous CLDN18 expression. [file HIS-86-640-s001.pdf]
