# Supplementary material for: Expression of claudin‐18.2 in cholangiocarcinoma: a comprehensive immunohistochemical analysis from a German tertiary centre
Source: Histopathology. 2024 Dec 27;86(4):640–6. doi: 10.1111/his.15407 (PMC11791722; doi:10.1111/his.15407)
Supplement: Supplementary file 4 — Table S1. Baseline characteristics. Positive M1 status reflects an intraoperative finding of M1 situation (e.g., distant lymph node metastasis) that was not known before surgery. [file HIS-86-640-s004.pdf]

Suppl. Table 1.

	VENTANA® CLDN18		
**Characteristics**	**Positive (n=21) No. (%)**	**Negative (n=139) No. (%)**	***p*-Value**
**Sex**			0.421
Female	9 (42.9)	47 (33.8)	
Male	12 (57.1)	92 (66.2)	
**Age at initial diagnosis**			0.777
Mean, years, (range)	65.9 (42-82)	65.2 (38-86)	
**ECOG**			0.088
0	10 (47.6)	94 (67.6)	
1	10 (47.6)	41 (29.5)	
2	1 (4.8)	4 (2.9)	
**CA-19/9** (ng/ml)			0.282
< 37	4 (19)	49 (35.3)	
≥ 37	10 (47.6)	63 (45.3)	
n.a.	7 (33.3)	27 (19.4)	
**Tumor size** (cm)			0.23
≤ 5	15 (71.4)	80 (57.6)	
> 5	6 (28.6)	59 (42.4)	
**Single Tumor**			0.277
Yes	16 (76.2)	89 (64)	
No	5 (23.8)	50 (36)	
**Pathological grade**			0.903
Grade 1	1 (4.8)	2 (1.4)	
Grade 2	14 (66.7)	100 (71.9)	
Grade 3	6 (28.6)	37 (26.6)	
**M status**			0.002
M0	16 (76.2)	132 (95)	
M1	5 (23.8)	7 (5)	
**LNM**			0.008
No	9 (42.9)	90 (64.7)	
Regional	8 (38.1)	44 (31.7)	
Distant	4 (19)	5 (3.6)	
**R status**			0.207
R0	14 (66.7)	106 (76.3)	
R1	7 (33.3)	28 (20.1)	
Rx	0 (0)	5 (3.6)	
**L status**			0.466
L0	9 (42.9)	66 (47.5)	
L1	10 (47.6)	51 (36.7)	
Lx	2 (9.5)	22 (15.8)	
**Pn status**			0.022
Pn0	2 (9.5)	43 (30.9)	
Pn1	17 (81)	72 (51.8)	
Pnx	2 (9.5)	24 (17.3)	
**V status**			0.512
V0	14 (66.7)	100 (71.9)	
V1	4 (19)	19 (13.7)	
Vx	3 (14.3)	20 (14.4)	
**Recurrence**			0.709

Yes	13 (61.9)	80 (57.6)	
No	8 (38.1)	59 (42.4)	
**Cholelithiasis**			0.065
Yes	3 (14.3)	6 (4.3)	
No	18 (85.7)	133 (95.7)	
**PSC**			0.335
Yes	0 (0)	6 (4.3)	
No	21 (100)	133 (95.7)	
**Viral hepatitis**			0.491
Yes	1 (4.8)	13 (9.4)	
No	20 (95.2)	126 (90.6)	
**Diabetes**			0.528
Yes	6 (28.6)	31 (22.3)	
No	15 (71.4)	108 (77.7)	
**Liver cirrhosis**			0.335
Yes	0 (0)	6 (4.3)	
No	21 (100)	133 (95.7)	
**LDH**			
< 248	13 (61.9)	69 (49.6)	0.036
≥ 248	1 (4.8)	37 (26.6)	
n.a.	7 (33.3)	33 (23.7)	
**Bilirubin**			0.573
< 1.4	13 (61.9)	92 (66.2)	
≥ 1.4	8 (38.1)	43 (30.9)	
n.a.	0 (0)	4 (2.9)	
